# The Development of a Biomimetic Design Tool for Building Energy Efficiency

**DOI:** 10.3390/biomimetics5040050

**Published:** 2020-10-12

**Authors:** Negin Imani, Brenda Vale

**Affiliations:** Wellington Faculty of Architecture and Design Innovation, Victoria University of Wellington, Wellington 6140, New Zealand; Brenda.Vale@vuw.ac.nz

**Keywords:** biomimetic design, sustainable design, biomimicry, thermal adaptation, thermoregulation

## Abstract

The initial aim of the research was to develop a framework that would enable architects to look for thermoregulation methods in nature as inspiration for designing energy efficient buildings. The thermo-bio-architectural framework (ThBA) assumes designers will start with a thermal challenge in a building and then look in a systematic way for how this same issue is solved in nature. The tool is thus a contribution to architectural biomimicry in the field of building energy use. Since the ThBA was created by an architect, it was essential that the biology side of this cross-disciplinary tool was validated by experts in biology. This article describes the focus group that was conducted to assess the quality, inclusiveness, and applicability of the framework and why a focus group was selected over other possible methods such as surveys or interviews. The article first provides a brief explanation of the development of the ThBA. Given the focus here is on its validation, the qualitative data collection procedures and analysis results produced by NVivo 12 plus through thematic coding are described in detail. The results showed the ThBA was effective in bridging the two fields based on the existing thermal challenges in buildings, and was comprehensive in terms of generalising biological thermal adaptation strategies.

## 1. Introduction

The literature review on biomimetic energy efficient building design undertaken prior to developing the thermo-bio-architectural framework (ThBA) showed architectural researchers were paying increasing attention to the solutions organisms use to adapt to their thermal environment, in the hope these could lead to more sustainable building [1]. It also seemed problem-based bio-inspired design (BID), where a designer starts with a challenge and then looks to biology, had been researched in engineering fields [2,3] but not yet fully in architectural design [4].

Attempts to develop a problem-based framework for bio-inspired design are: Biomimicry 3.8 led by Benyus, BioTriz led by Julian Vincent [3], ‘Biomimetic for Innovation and Design Laboratory’ led by Li Shu [2], ‘Design & Intelligence Laboratory’ led by Goel, and ‘Plants Biomimetic group’ led by Thomas Speck [5]. However, none of these focused on sustainable building design. Recent research by Martín-Gómez et al. [6] used a solution-based rather than problem-based approach to bio-inspired energy efficient building design, but this seemed less useful as architectural design is problem-based. Earlier Badarnah [7] developed a methodology for the problem-based BID approach but this was not developed into a tool. Her research stressed the necessity of developing an optimal classification scheme accepting the fact that there could be various ways of categorising biological information.

Although the biology-based design approaches listed above sound useful, they all suffered from a lack of clarification in the exploration and investigation stage. Given energy use reduction is a building design problem, a problem-based approach would seem ideal. This means that once architects are aware of thermal issues or need to enhance the thermal performance of a building using BID strategies, the immediate aim is to find relevant biological examples. Unfortunately, there is no method for conducting this search. The ThBA attempted to fill this gap.

## 2. Materials and Methods

Energy efficient building design is affected by the limitations and opportunities of the project in hand and the resultant architectural design strategies. The ThBA was intended to give architects the ability to find biological solutions relevant to the thermal characteristics and requirements of buildings. When upgrading an existing building, its current energy performance is normally the starting point for any design, and this suggested improving the thermal performance of an existing building would be a basis for developing and testing the ThBA.

The first step was thus to articulate the thermal issues in a number of existing office buildings as case studies. This was important as in a problem-based approach the bio-inspired design process begins with design challenges. These need to be connected to solutions offered by biological organisms in a systematic way, in a process known as design by analogy [8,9]. Given this, the plan was the ThBA would have two sides relating to the distinctive fields of biology and architecture with links between through which the analogies identified from the natural world and their corresponding thermal adaptation principles could be transferred.

For the architecture side of the ThBA, the types of thermal challenge were identified through running energy simulations and comparative energy analysis of sample office buildings [10]. The biology side of the ThBA was created by means of a comprehensive literature review of heat transfer mechanisms in biophysics. This also gave insight into ways to categorise the biological thermoregulation strategies and established the basis of the overall structure of the biology side of the ThBA.

Because the ThBA was a cross-disciplinary tool and was developed by the first author with her expertise in architectural design, it was necessary it was evaluated by biologists to ensure the categorisation and systematised structure of the ThBA was effective and inclusive in terms of its generalised thermal adaptation strategies. The latter was important as the literature had questioned the possibility of generalising the thermal adaptation strategies of all types of plants and animals. Also, recent research in this field [7] stated that any trial gathering of biological data would require a comprehensive biological literature review as well as involving biologists from diverse fields in the research process.

Developing the ThBA required classifying and generalising the thermal regulation strategies used by living organisms. This was achieved through means of a comprehensive biological literature review. How this was done is briefly explained in Section 2.1.

### 2.1. Method Part 1: Literature Review

The literature review was conducted in two steps. The first step was to understand methods of heat transfer in nature. Reviewing the literature on the heat transfer methods and thermal adaptation strategies of organisms failed, however, to offer a categorisation scheme. The thermal adaptation mechanisms were also scattered and not classified in a way that could be useful for architects. This led to a second step that involved investigating the key themes of thermal physiology as suggested in the original glossary of thermal physiology revised by the Commission for Thermal Physiology of the International Union of Physiological Sciences (IUPS) [11]. Thermal physiology concerns the laws around the regulation of heat [12].

#### 2.1.1. Thermal Physiology

As the literature review did not reveal a hierarchical classification of thermal regulatory strategies, the thermal physiology of heat regulation was studied to create a foundation for categorising thermal adaptation strategies. The themes come from the 479 terms in the UIPS glossary [11]. This was then reinforced by the meanings of ectothermy and endothermy which informed the classification of thermal adaptation mechanisms into behavioural and autonomic mechanisms.

To reduce misinterpretation, this research used the many terms on which the experts have agreed [10]. Table 1 briefly introduces the key physiological terms and their definitions that were used to assist the categorisation of thermal adaptation strategies.

#### 2.1.2. Analysis and Results of Method Part 1

Among the thermal physiology terminologies suggested by the IUPS, the most appropriate themes for structuring the architectural side of the ThBA were (1) endothermy and ectothermy and (2) the involuntary (autonomic or physiological) and voluntary (behavioural) aspects of thermoregulation. For the former, the main difference between endotherms and ectotherms is the location of the heat sources used for thermal adaptation. Generating heat in the body of endotherms is equivalent to heat generation by heating systems. Unlike endotherms, ectotherms use behavioural adaptation strategies to regulate their body temperature. The equivalent in architecture would be placing bedrooms on the east side of the house so they warm up in the early morning sun to achieve a higher indoor temperature before getting up.

Autonomic or physiological thermal adaptation strategies are achieved through or associated with physiology or physiological changes in the bodies of organisms. These are called active thermoregulatory mechanisms. Behavioural thermal adaptation strategies, on the other hand, do not cause any changes in the body of an organism or relate to its physiology. Thermoregulation in this state happens through behaviour, and such behaviours are known as passive thermoregulatory strategies.

Picturing buildings as living organisms, a sustainable building design strategy is active when it changes in some way. This means there is a change in a building system, such as the automatic opening of roof windows to vent hot air, which parallels the way physiological changes occur through alterations in circulatory systems of the bodies of organisms. On the other hand, passive design strategies do not make any changes to a building or involve heating and cooling circulatory systems but rather concern an independent parameter changing either inside the envelope, or outside the building. This is similar to the changes in the behaviour of organisms. Example of these are provided below.

For the concepts of endothermy and ectothermy, thermal adaptation strategies are active when the heat is generated in the body of an organism and are seen as passive when the heat source is outside the body of an animal or plant. This suggested a parallel as a building can adapt to its environment through both passive and active design strategies. These were the ideas used to organise the ThBA.

##### Active Methods of Thermal Adaptation in Organisms

Countercurrent heat exchange is an active thermoregulation strategy used by many birds (e.g., some penguins) and mammals (e.g., reindeer) that enables them to transfer heat to parts of their bodies. For instance, the warm arteries in the legs lose heat to the veins taking the blood back to the heart to avoid excessive heat loss through the feet.

##### Active Methods of Thermal Adaptation in Buildings

One of the building parallels to autonomic thermoregulation in organisms would be a smart system in which the internal temperature is regulated without the need for human intervention. For example, controlling solar gain through liquid-shielded windows contributes to a reduction in the thermal load [14]. Water circulation in windows can also reduce unwanted heat loss and heat gain [15]. Another example could be an active solar system collecting solar energy, transferring it in the form of heat to either air or liquid, and distributing the latter through pumps or fans.

##### Passive Methods of Thermal Adaptation in Organisms

Almost all behavioural strategies related to endothermy and ectothermy are passive. For example, burrows play an important part in the acclimatisation of animals to an environment, being the thermally buffered microclimates some animals use to survive [16]. The important issue is the animal physiology remains unchanged, making it passive thermal adaptation.

##### Passive Methods of Thermal Adaptation in Buildings

An example of passive thermal adaptation in buildings is solar heat gain through glazing or skylights. Buildings in these states behave like ectotherms. Thermal migration behaviour in ectotherms, for example moving into the sun in the early morning, is like having spaces in a building with different temperatures with people moving between these as necessary.

This categorisation of biological thermal adaptation strategies as active and passive was then used as a means of seeking analogies in architecture and finally informed the primary structure of the ThBA. Accordingly, for each mechanism, parallels in energy efficient building design were introduced, and these were used for the architectural side of the ThBA.

### 2.2. Method Part 2: Focus Group

This research used qualitative data collection to validate the ThBA in the form of a focus group of biologists. The term “focus group” has been associated with market research and it has also been referred to as “group discussion” by social researchers who work with academic and applied research studies [17]. Focus groups in general are more about prioritisation so as to facilitate decision making [18]. Group discussions are usually used to generate critical comments [19], to yield insights, and to enable the creation of ideas [20].

Focus groups also enable the amalgamation of multiple viewpoints as a number of participants engage in a conversation around a topic at the same time. In addition, the researcher can have the opportunity contribute more actively in the topic analysis and reasoning [21], and to hear divergent opinions [22], which can sometimes lead to unexpected results [23]. However, the aim here was neither to seek the opinions of participants on a particular issue nor the reasons behind survey results, but rather to seek agreement of participants on the comprehensiveness of the ThBA, as it is possible to achieve consensus using a focus group [24].

Even though the Delphi method is more useful for achieving group consensus [25,26], this seemed an unnecessary complication as the goal was to make sure that no thermoregulatory method found in nature was missing from the ThBA rather than asking experts what it should contain.

Focus groups have been used for evaluating user-centered tools, although this usually means investigating usability, and applicability of the relevant applications or systems [27]. In some human-computer interaction research, the key point has been ensuring the user’s full comprehension of the tool and its structure [28], so ensuring biologists understood the ThBA was important, as well as checking that nothing was missing from it. Given the ThBA was a design tool, group discussions seemed to be a good fit as focus groups are extensively used in situations where design has been recognised as the central issue [17]. Also, a focus group gave the lead researcher the opportunity to describe the complex structure of the ThBA and the reasons behind it [29]. Getting the input of biological knowledge from professionals needed a series of open-ended questions. Individual interviews and responding to direct questions does not necessarily provide access to people’s knowledge [21]. Instead, focus groups are a suitable technique for brainstorming [30] as questions can be framed in a clear way to ease group discussion [31].

Since the ThBA was developed to be used by architects, only its biology side was evaluated by the focus group. A comprehensive understanding of the ThBA configuration and the logic behind its classification of the biological solutions, should have created a mental map for the experts. With a clearer mental map, the hope was it would be easier for biologists to comment on any missing information and the appropriateness of the hierarchical structure. It was also important for the expert focus group to discuss whether the thermal adaptation strategies were correctly generalised and systematically branched into sub-categories leading to examples of organisms. The aim was that consensus would emerge from a collective procedure rather than from individual opinions [32].

At an earlier stage before developing the ThBA, this research was presented in the Ecology and Evolution seminar series 2018 in the School of Biological Sciences. The purpose of this was to ensure the language used for the explanation of the ThBA interdisciplinary framework was appropriate. As with other interdisciplinary research, successful communication between disciplines increases the chance of receiving trustworthy and useful results. Audience comments at the seminar suggested the research question was well-worth investigating, and a number of those attending were interested in taking part in any future focus groups.

#### 2.2.1. Sample and Sizing

It has been argued that there is no limit to the number of participants in a focus group and the sample size is determined based on the aim of the project [21]. Others say the size of the ideal focus group is limited, and can vary from 4 to 14 [33], although Morgan [24] and Bloor et al. [34] suggest between 6 and 10.

Participants in the focus group were identified by their area of expertise and invited to the focus group through emails. From the 50 emails sent, 20 experts were interested in taking part but had limitations with their schedules. Around 80% of those who expressed an interest in participating were either professors or associate professors. Eventually, seven participants agreed to take part with one pulling out at the last minute because of a schedule change. The group members consisted of three PhD students, and three academics none of whom had a supervisory relationship with any of the PhD students.

The research suggested there were eight major biological fields of which six were branches of zoology including Entomology (the study of insects), Herpetology (the study of reptiles and amphibians), Ichthyology (the study of fish), Malacology (the study of molluscs), Mammalogy (the study of mammals), and Ornithology (the study of birds), as well as botany and microbiology. The final participants covered a range of expertise including botany, and marine and terrestrial animal biology. Also, there was an approximately equal distribution of expertise between those working on vertebrates and invertebrates at different biological scales from cells to tissues, organs, and single organisms.

#### 2.2.2. Questionnaire and the Three Parts of the ThBA

**Figure 1:**
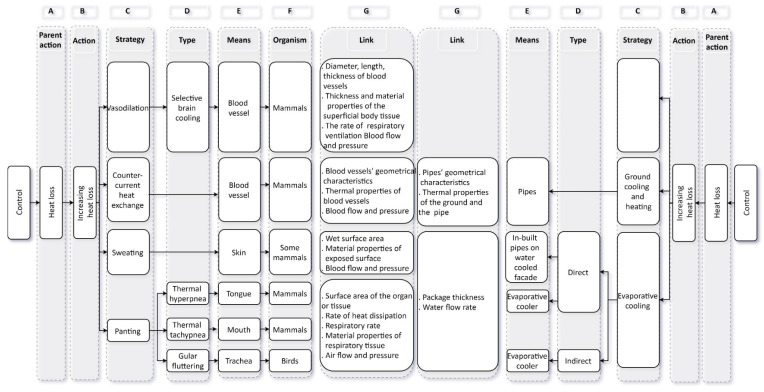
Section of the Thermo-bio-architectural Framework (ThBA) (part of the focus group materials) related to active strategies for animals.

Participants were provided with a list of questions and an introduction that included a brief explanation of the ThBA structure, and the motivation behind its development, as well as guidance on how to use it. Each pair of participants shared a copy of the three parts of the ThBA. Two parts were related to passive and active strategies for animals and one part for strategies used by plants. The latter had only one part since the main passive and active strategies as provided by IUPS did not match properly with the thermoregulatory mechanisms used by plants. Figure 1 shows a section of the ThBA related to active strategies for animals. Each side of the ThBA consists of different columns, the order and content of which are explained below:

In the complete version of the ThBA column A consists of three main branches called ‘parent actions’. These are heat loss, heat gain, and heat generation. As Figure 1 is only part of the ThBA only heat loss strategies are shown. Column B contains the three sub-branches of ‘parent actions’. These are increasing heat loss, decreasing heat loss, and avoiding heat loss and only the first part is shown in Figure 1. For each ‘action’ (column B), relevant ‘strategies’ are presented in column C. The strategies then branch into ‘types’ (column D). For each ‘type’, there is a ‘means’ that refers to the parts of animals or plants responsible for thermal adaptation. Columns F introduces examples of organisms using that strategy. In the design-by-analogy process, a link should be made between source and target concepts to enable knowledge transfer from the biology domain into architecture through a series of parameters. These are presented in column G.

The architecture side of the ThBA shows sustainable building design strategies presented under the same columns except for column F as the analogy between a building and an ‘organism’ made column F irrelevant on the architecture side. Empty boxes were used where there was no parallel design strategy to an existing biological thermal adaptation mechanism. For example, ‘vasodilation’ did not have a parallel in architecture.

As shown in Figure 1, sweating and panting are analogous to evaporative cooling. Indirect evaporative cooling is parallel to gular fluttering as one type of panting, while thermal hyperpnea, thermal tachypnea, and sweating are equivalent to direct evaporative cooling. The links documented for each type can be used for translating thermoregulatory principles in sweating and panting to those contributing to the function of evaporative coolers. The ThBA lists the key parameters required for transferring mechanisms from biology to architecture. For example, in thermal tachypnea, the respiratory parameters, air sac properties, and cooling efficiency of panting in birds affect their panting mechanism [35].

#### 2.2.3. Analysis of Method Part 2

This research used NVivo 12 plus and latent content analysis for coding and theme identification to allow the interpretation of the hidden layers under the text [36,37]. The audio record was transformed into text using Trint. The text was abstracted and codes, themes, and categories were used to aggregate the data.

A thematic approach was used for the data analysis based on the questions and the narratives of participants. To create a basis for the most common themes, transcriptions were read several times, and the text was then imported to NVivo 12 plus for further coding and more detailed analysis. Codes were grouped into different categories, then clustered in a category shared commonality, and finally themes were created to link similar categories.

The following sections introduce the themes identified including their categories and subcategories. The main reason for the focus group was to evaluate the applicability, usability, and inclusiveness of the data categorised and aggregated in the ThBA framework. Accordingly, the main themes were to some extent predictable due to this purpose.

#### 2.2.4. Development of Themes and Sub-Themes

The themes were derived out of the concerns raised during the research. The focus group was set up to ensure

1. the participants understood the framework,
2. they had evaluated the ThBA and;
3. what the researcher had achieved in developing the framework was valid.

Accordingly, there were three sets of questions, each defined to address the concerns mentioned above.

Given ‘understanding the ThBA’ was the first theme, it was important the participants fully understood both the framework and the purpose behind its creation. This was targeted in the discussion by asking questions or talking about the framework. However, no question was directly asked to confirm whether the participants had understood the ThBA. For the second theme, ‘framework evaluation’, the main purpose of the focus group was to examine the contents of the ThBA.

A series of concepts seemed to play a critical role in the context of thermal adaptation principles in the understanding of the nature-biology relationship. These concepts were revealed during the exploration of the ThBA structure which formed the third theme centred around the validity of the ThBA. It was not only important to evaluate if the ThBA accurately reflected how thermal challenges are dealt with in nature, as this formed its basis, but also to examine the extent to which this knowledge had been successfully incorporated into its structural organisation. The third set of questions provided the opportunity to learn whether these goals had been achieved.

Given these three themes had to be addressed the text was analysed against them. The coding process was performed using the whole text rather than scanning through the answers given for individual questions. Initial lists of codes were created based on the topics brought up by biologists [38]. The codes achieved through descriptive coding were then reviewed and grouped under a category heading. These groupings were based on their similar patterns and commonalities as well as their relationship to the themes. The categorisation of codes was repeated multiple times, each iteration being labelled with an analytical code. The process of re-categorisation continued until the remaining categories no longer shared any commonalities and hence could not be merged into a new group or labelled with an analytical code. The last sets of codes were the subthemes falling under the main themes.

Almost all codes fell into at least one theme, and only five were identified as emergent topics unrelated to any of the themes. These codes were amalgamated under a new theme labelled ‘suggestions’.

**Figure 2:**
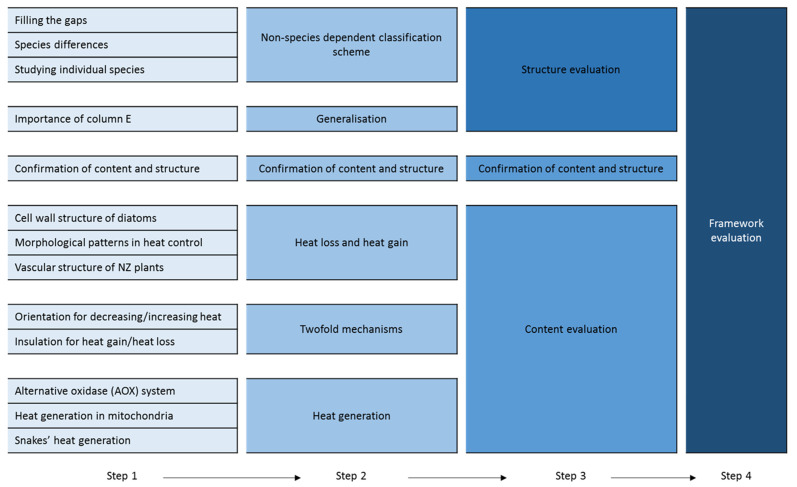
Different stages of coding based on the second theme ‘framework evaluation’.

Figure 2 shows the different stages in which the codes were generated and grouped under the second theme of ‘framework evaluation’. A similar approach was used to derive the sub-themes of the first and the third themes. The light blue boxes represent the initial codes and the blue gets darker each time the codes group under a new category. This continued until the themes and sub-themes were defined.

**Figure 3:**
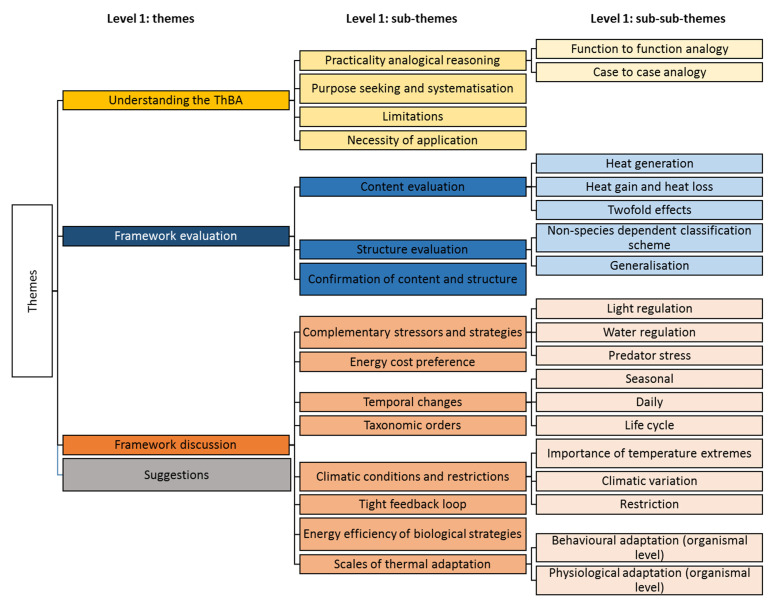
The coding structure including the main emergent themes and sub-themes.

The themes in Figure 3 are shown in different colours. The gradient for each colour represents the hierarchy as it gets lighter in each level from the top (main themes) to the bottom (sub-themes and sub-sub-themes).

**Figure 4:**
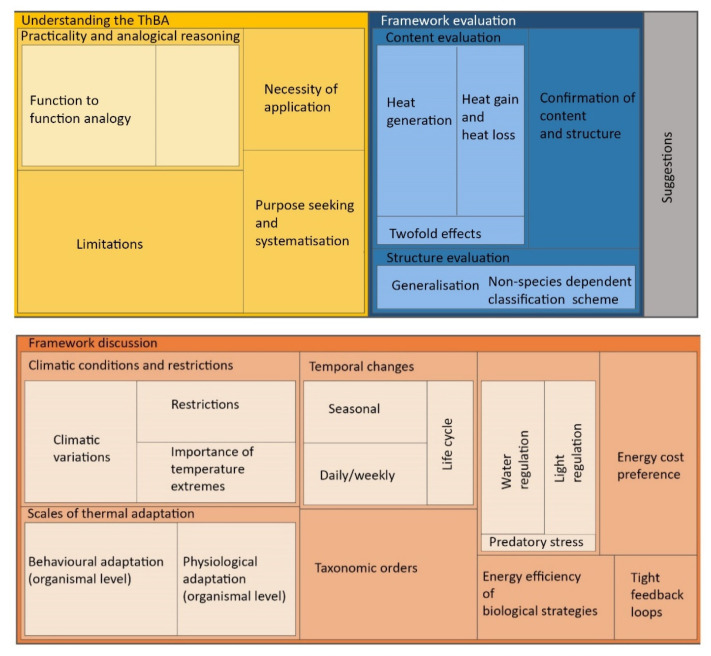
Proportional representation of all references coded for the main themes.

Figure 4 shows the hierarchical structure of data with the highest level showing the four main emergent themes of:

1. Understanding the ThBA,
2. Framework evaluation,
3. Framework discussion, and
4. Suggestions.

**Figure 5:**
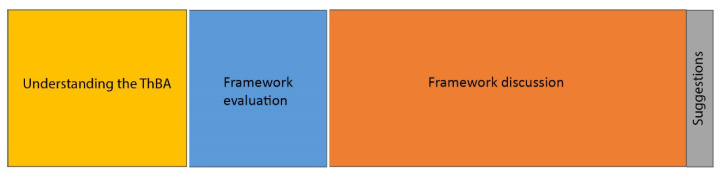
Proportional representation of all references coded for the main themes.

The size of the rectangles in Figure 5 represents the total number of references coded for the four main themes. The result showed the balance between the two parts of:

1. Understanding the ThBA and framework evaluation
2. Framework discussion and suggestions.

The dominance of the second part underlines the success of the ThBA, as the time spent on participant understanding and approval of the ThBA was noticeably shorter. It also highlights the validity of the concepts, and the level of biological knowledge and its use within the ThBA to make it appropriate for architects and building designers.

## 3. Results

The analysis showed that the ThBA could perform properly in its current configuration without the need to add extra layers to its structure. Furthermore, the understanding of the biological concepts was confirmed as valid and it was not thought that a rearrangement was necessary. One suggestion for change was raised but it was made clear that this could be added to the framework in the next stage of this research.

The following sections detail the findings in relation to the themes but first, the participant contributions are visualized.

### 3.1. Visualisation of the Participant Contributions

**Figure 6:**
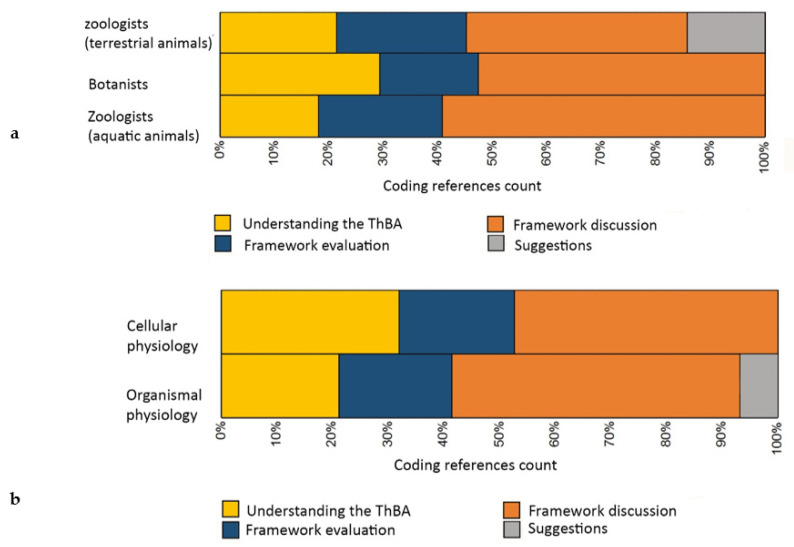
Distribution of contributions based on (**a**) participant expertise, (**b**) biological scale under focus.

Figure 6a,b indicate the percentage contribution made by each participant to the emergent themes. Figure 6a breaks the contribution down by specialism, and Figure 6b by whether the participant worked at the scale of organism or cells.

The following sections briefly explain the subtheme results.

### 3.2. Understanding the ThBA

Four points emerged from the focus group discussion.

1. why the ThBA has been developed (purpose seeking) and that a bottom-up approach to the BID process could be a system for designing energy-efficient buildings (systemisation of the BID process);
2. why the ThBA should be used (need for its application);
3. how the ThBA could be utilized (practicality and analogical reasoning) and;
4. where the ThBA might not be useful (its limitations).

These showed the ThBA was understood by participants. Aspects of this understanding are briefly explained below.

Purpose seeking and systematisation: Participants discussed metaphorical uses of biological functions and processes in biomimetic architecture. Part of the discussion was also concerned the effectiveness of empty boxes in the ThBA and the fact these point to gaps in architectural design.

Necessity for the ThBA application: One of the concerns was about New Zealand buildings not being adapted to their climatic conditions. An example was designing large windows to enhance daylighting without considering their effects on increasing internal temperature. In addition, participants felt that architects as amateur biologists do not consider the whole effect of using a biological strategy, as not all organisms are efficient in their use of energy.

Practicality and analogical reasoning: The analogy between nature and architecture was made at two scales.

The larger scale dealt with the relationship between a building and its environment, which is similar to an organism and its environment. There was also debate about whether buildings should be recognised as ectotherms rather than endotherms. Heat generation in buildings was linked to endothermy, and this seemed to be the reason why architects are more interested in animals as biological inspiration.

Biologists referred to animals as clever organisms in terms of solving thermal problems compared to plants, but they felt buildings were structurally similar to plants. They noted that ectotherms and plants could survive in harsh climates because their body temperature stays at the same level as their environment. The capability of organisms in temporarily stopping activities was a strength compared to endotherms. At a smaller scale, there were analogies between building and organism adaptation strategies.

Limitations: All participants agreed the constraints in architecture as well as the requirements of construction often fall short of the ideal espoused by biomimicry.

**Figure 7:**
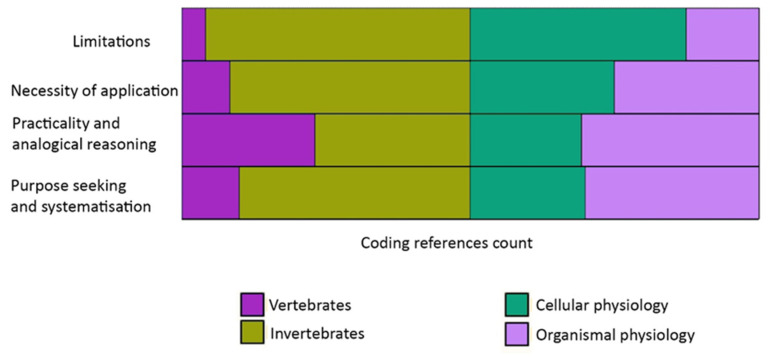
Spread of codes based on expertise of participants.

Outline: As shown in Figure 7 and Table 2, expertise fell into three categories based on the classifications/levels/scales of participant specialisations. These categories were:

(a) Terrestrial and marine zoology, and botany,
(b) Organism and cellular physiology; and
(c) Vertebrates and invertebrates

Figure 7 presents the proportional contribution of participants based on b and c. The codes generated, covered cellular and organism physiology almost equally while that linked to invertebrates was more dominant.

Table 2 shows that most codes were related to practicality and analogical reasoning. Botany made the highest contribution with most codes generated for the ‘limitations theme’, which is interesting regarding the discussion over whether buildings were more like plants or animals.

### 3.3. Framework Evaluation

The ThBA framework evaluation had two parts:

1. Content assessment
2. Structure assessment.

The following explains the minor changes participants felt could be considered for both the content and structure of the ThBA.

Content assessment: A few strategies related to heat generation mechanisms were missing. However, some strategies suggested by participants were in the framework but were categorised differently. For example, participants stated shivering as a heat generation mechanism in snakes was a passive strategy while it was already classified under active strategies.

A new strategy mentioned was a biochemical activity occurring at the cellular level in plant respiration. Another was heat generation in swordfish that takes place in mitochondria and red muscles. The biologists also suggested stationary organisms, especially those underwater, cannot control their temperature and thermal adaptation is carried out at a cellular level. They thought it was not necessary to add these to the ThBA as the cellular level was not within the scope of this research.

Experts discussed how the structural organisation and morphological features of some organisms contribute to heat gain. Examples were the specific vascular architecture of some New Zealand plants, the shells of intertidal bivalves, and the cell wall structure of diatoms. In all three, light focusing capability is used for photosynthesis, which increases the temperature. Morphological characteristics leading to thermoregulation were already documented in the ThBA but investigating such patterns in organisms was beyond the scope of this research.

Structure assessment: Confirmation of the ThBA structure was achieved as participants accepted its

(a) generalisation and
(b) non-species dependent classification scheme.

Confirmation of generalisation was achieved through documenting participant quotes according to the contexts in which the discussion occurred.

For non-species dependent classification, biologists thought climatic variation might trigger the evolution of different endotherms and ectotherms, but they also felt that the principles of thermoregulation would be similar. In addition, the importance of species investigation was discussed. This is found in bioprospecting where researchers study the biochemistry of small and nondescript marine animals to find pharmaceutical antibiotics.

Outline: Table 3 illustrates the percentage breakdown of the codes generated for the ThBA content and structure evaluation based on expertise, showing that the academics produced more quotes related to the structure of the ThBA, while PhD students were more active in the content analysis.

In the structure evaluation column, fewer codes were linked to cellular physiology with the opposite for the content evaluation column, where organism physiology was recorded fewer times. Structural evaluation received no input from marine zoology. Both content and structural evaluation were endorsed by experts in terrestrial zoology and botany (Table 3).

### 3.4. Framework Discussion

Table 4 shows the subthemes that emerged for the third theme ‘framework discussion’. This highlighted the validity of the ThBA. However, two unexpected subthemes occurred during the data analysis.

In contrast to earlier research looking to animals for bio-inspired buildings [39,40,41,42], the experts in biology discussed the similarity between buildings and plants. This change in focus could open a new window for further research. Another unexpected outcome was the collective agreement of biologists that not all strategies in nature are energy efficient. This avenue is opposed to common beliefs about sustainability in nature. Non-energy efficient biological strategies might not be useful for energy-efficient building design.

### 3.5. Suggestions

None of the themes introduced above concerned significant modification of the ThBA framework. One participant discussed including climatic classification within its structure. Participants believed that the ThBA should not be changed but did see that climatic classification could be added to the framework in the future.

## 4. Discussion

The focus group results proved the initial hypothesis that developing a systematic process for designing energy-efficient bio-inspired buildings was possible. Reviewing the results against the themes revealed the participants had a good understanding of the ThBA and its application in building design. The framework contents and structure were approved, and no strategy was left out.

The findings of the focus group study are summarised below:

- The ThBA was confirmed as including almost all thermal adaptation strategies, and all those that could be immediately useful. Generalising thermal adaptation mechanisms was seen as possible by the experts as they believed such strategies are usually common for endotherms and ectotherms, although they also said climatic variation might lead to the evolution of different species.
- Participants gave examples and were able to link nature to architecture by referring to a series of analogies. They discussed how buildings could be seen as more like plants than animals.
- All participants agreed there is a limitation in building design if the aim is to imitate thermal adaptation strategies found in nature. Buildings cannot shut down temporarily. This is in contrast with the temporal changes in organisms that might occur on a daily, weekly, or annual basis.
- Participants believed that many buildings not environmentally well-adapted are being used for decades. This is in contrast with what happens in nature as organisms die if they do not fit their environment. They added it is not energy efficient to destroy buildings that do not adapt to their surroundings.
- The participants emphasised how not all biological thermal adaptation strategies are energy efficient. The group also agreed there is an energy cost for thermal adaptation strategies and organisms compromise over this all time.
- Exploring individual species or bio-prospecting was mentioned as something recently used for producing pharmaceutical products. However, searching for non-documented species was suggested only if architects could not find a solution in nature from the existing database. Otherwise, the ThBA coverage and structure should support queries related to heat control.
- The structural hierarchy and the classification scheme were seen as useful by all participants, and no reconfiguration of the data was advised.
- The participants agreed thermal adaptation strategies take place at different scales. They also believed what happened at different scales was not necessarily linked, and looking into each scale would reveal a different solution.
- The group referred to other environmental stressors being in close relationship with thermal stressors to the extent that responding to one of them would result in thermal adaptation. The stressors mentioned were light, water, nutrients, and predators. This might not be relevant to buildings.
- It was felt organisms native to one climate might offer strategies in another climate, meaning that building designers should not limit themselves to the local environment when looking for analogous biological solutions. The ThBA was therefore useful as it was not organised based on the temperature gradients to which organisms adapt.

## 5. Conclusions

Reviewing thermoregulatory states, passive and active ways of thermoregulation linked to the concepts of endothermy and ectothermy emerged as the most appropriate categorisation for the ThBA. This is because the concepts of endothermy and ectothermy had exact parallels in energy efficient building design. The resultant ThBA was used in the form of three diagrams in a focus group panel of biological experts to evaluate its inclusiveness and usability. This expert focus group confirmed the biology side of the thermo-bio-architectural framework and approved its applicability in terms of categorising biological thermal adaptation strategies based on thermal needs. They confirmed the thermoregulation strategies were comprehensive and represented an inclusive list of biological organisms. This also showed the areas where sustainable building design cannot replicate biological thermoregulatory mechanisms. It also appeared that some biological thermoregulation strategies are energy-consuming, making thermoregulation in buildings and organisms incompatible in some aspects, although this could be due to the limitations in the current ways of designing sustainable buildings.

## Figures and Tables

**Table 1:** Key physiological terms.

Acclimation and Acclimatisation	Acclimation is the physiological and behavioural changes that occur within the bodies of organisms so they can endure immediate environmental stressors. Acclimatisation, on the other hand, refers to changes that take place in the body during the lifetime of organisms and as a response to natural climatic conditions.
Timeframes in adaptation	Crepuscular refers to adaptation strategies that take place at dusk or dawn while nycthemeral indicates the occurrence of these on a 24 h basis.
Feedback loop control	This forms the backbone of homeostasis, which is the ability of an organism to maintain equilibrium and so to stabilise the effect of a changing variable.
Thermal regulation	This identifies the process by which organisms adapt to temperature as an environmental stressor.
Autonomic and behavioural thermal regulation	Autonomic thermoregulation is the regulation of body temperature through involuntary responses to thermal stressors. Behavioural thermoregulation requires the coordinated movement of an organism towards a more favourable thermal environment.
Normothermy, hyperthermia, hypothermia, cryothermy	These are thermoregulatory states pointing to different conditions of the body temperature as either within normal limits (normothermy), above the range (hyperthermia), or below of a species in a normal state (hypothermia). Cryothermy is where the body temperature falls below the freezing point of the body tissue.
Homeothermy, heterothermy, and poikilothermy	These are thermoregulatory patterns of the temperature variations that might occur within defined limits except for conditions where the ambient temperature varies greatly (homeothermy), beyond the boundary of that of homeothermy (heterothermy), or over a broad range (poikilothermy).
Eurythermy and stenothermy	The tolerance of organisms to the range of environmental temperature is known as eurythermy when the range is wide and stenothermy when it is narrow.
Heat and cold tolerance	The ability to tolerate high and low ambient temperatures comprising a variety of physiological properties.
Avoiding thermal stress	The mechanisms animals use to get away from the environment in which they are under thermal stress through avoiding the space or doing normal activities [13].
Regulating thermal stress	This occurs through a combination of behavioural and physiological changes in the bodies of organisms.
Conforming to thermal stress	The mechanisms whereby animals undergo changes in their physiological and biochemical levels so they can function at a very low level without undergoing huge changes.
Endothermy and ectothermy	The body temperature of ectotherms (cold-blooded animals) follows that of their immediate environment while endotherms (warm-blooded) maintain a near constant body temperature.

**Table 2:** Spread of codes based on field of expertise.

	A Limitations	B Necessity of Application	C Practicality and Analytic Reasoning	D Purpose Seeking and Systematisation
Zoology (terrestrial)	1	1	6	1
Botany	9	4	7	3
Zoology (marine)	2	1	0	1

**Table 3:** Percentage breakdown of the codes generated for the content and structure evaluation.

	Content Evaluation	Structure Evaluation	
Zoology (terrestrial)	50%	50%	100%
Botany	60%	40%	100%
Zoology (marine)	100%	0%	100%
Cellular physiology	85%	15%	100%
Organism physiology	55%	45%	100%

**Table 4:** Emergent subthemes from the third theme.

	Subthemes	Sub Subthemes
concepts revealed during exploration of the ThBA	Complementary stressors and strategies	Light Water Nutrient regulation
	Temporal changes	Life cycle Daily/weekly Seasonal
	Climatic conditions and restrictions	Climatic variations Restrictions Importance of temperature extremes
	Scales of thermal adaptation	Behavioural Physiological
	Taxonomic orders or tree of life	
	Tight feedback loops	
Unexpected findings	Energy efficiency of biological strategies	
	Cost benefits of thermoregulation	
	Buildings are more like plants than animals	
